# Assessment of a Noninvasive Exhaled Breath Test for the Diagnosis of Oesophagogastric Cancer

**DOI:** 10.1001/jamaoncol.2018.0991

**Published:** 2018-05-17

**Authors:** Sheraz R. Markar, Tom Wiggins, Stefan Antonowicz, Sung-Tong Chin, Andrea Romano, Konstantin Nikolic, Benjamin Evans, David Cunningham, Muntzer Mughal, Jesper Lagergren, George B. Hanna

**Affiliations:** 1 Department Surgery & Cancer, Imperial College London, United Kingdom; 2 Institute of Biomedical Engineering, Imperial College London, United Kingdom; 3 Department of Oncology, Royal Marsden Hospital, London, United Kingdom; 4 Department of Surgery, University College London Hospital, United Kingdom; 5 Department of Molecular Medicine, Karolinska Institutet, Stockholm, Sweden; 6 School of Cancer and Pharmaceutical Sciences, King’s College London, United Kingdom

## Abstract

**Importance:**

Early esophagogastric cancer (OGC) stage presents with nonspecific symptoms.

**Objective:**

The aim of this study was to determine the accuracy of a breath test for the diagnosis of OGC in a multicenter validation study.

**Design, Setting, and Participants:**

Patient recruitment for this diagnostic validation study was conducted at 3 London hospital sites, with breath samples returned to a central laboratory for selected ion flow tube mass spectrometry (SIFT-MS) analysis. Based on a 1:1 cancer:control ratio, and maintaining a sensitivity and specificity of 80%, the sample size required was 325 patients. All patients with cancer were on a curative treatment pathway, and patients were recruited consecutively. Among the 335 patients included; 172 were in the control group and 163 had OGC.

**Interventions:**

Breath samples were collected using secure 500-mL steel breath bags and analyzed by SIFT-MS. Quality assurance measures included sampling room air, training all researchers in breath sampling, regular instrument calibration, and unambiguous volatile organic compounds (VOCs) identification by gas chromatography mass spectrometry.

**Main Outcomes and Measures:**

The risk of cancer was identified based on a previously generated 5-VOCs model and compared with histopathology-proven diagnosis.

**Results:**

Patients in the OGC group were older (median [IQR] age 68 [60-75] vs 55 [41-69] years) and had a greater proportion of men (134 [82.2%]) vs women (81 [47.4%]) compared with the control group. Of the 163 patients with OGC, 123 (69%) had tumor stage T3/4, and 106 (65%) had nodal metastasis on clinical staging. The predictive probabilities generated by this 5-VOCs diagnostic model were used to generate a receiver operator characteristic curve, with good diagnostic accuracy, area under the curve of 0.85. This translated to a sensitivity of 80% and specificity of 81% for the diagnosis of OGC.

**Conclusions and Relevance:**

This study shows the potential of breath analysis in noninvasive diagnosis of OGC in the clinical setting. The next step is to establish the diagnostic accuracy of the test among the intended population in primary care where the test will be applied.

## Key Points

**Question:**

What is the diagnostic accuracy of a breath test for esophagogastric cancer?

**Findings:**

In a multicenter diagnostic study of 335 patients, including 172 patients with esophagogastric cancer, the breath test demonstrated good diagnostic accuracy.

**Meaning:**

This study suggests the potential of breath analysis as a noninvasive tool in the diagnosis of esophagogastric cancer.

## Teaser

This multicenter validation study examines the accuracy of a breath test that analyzes volatile organic compounds for the diagnosis of esophagogastric cancer among adults in a curative treatment pathway.

## Introduction

In the United Kingdom, upper gastrointestinal symptoms account for at least 3% of consultations in primary care.^1^ The national esophagogastric cancer (OGC) audit reported 7044 cases of OGC diagnosed in 2016. Many patients present with advanced-stage disease and only 38% of cases can be treated with a curative intent.^2^ Current UK referral guidelines for suspected OGC focus on alarm symptoms such as dysphagia and odynophagia, despite these symptoms having poor sensitivity and specificity for OGC and often only occur in advanced disease translating into a poor outcome and overall survival.^3^ There is a wide range in the rate of oesophagogastro duodenoscopy (OGD) among general practice populations in England and OGC patients belonging to practices with the lowest rates of OGD referral are at greatest risk of poor overall survival owing to advanced tumor stage at diagnosis.^4^ Furthermore, OGD is an expensive invasive investigation, with poor uptake in specific ethic minority populations consequently affecting survival.^5^ This high prevalence of upper gastrointestinal symptoms coupled with the low incidence of OGC and the nonspecific nature of symptoms in early disease highlight the need for a triage test to direct patients to have OGD.

Volatile organic compounds (VOCs) emitted from the human body have been of interest to researchers for several decades,^6^ with associations previously suggested between specific VOCs and breath and lung, bladder, and breast cancers.^7,8,9^ We analyzed exhaled breath samples using selected ion flow-tube mass spectrometry (SIFT-MS) from 210 patients, 81 with OGC and 129 control patients. A diagnostic model of 13 VOCs was able to diagnose OGC with a sensitivity of 89% and specificity of 94%.^10^ Several phase 1 biomarker studies linking noninvasively measured VOCs to the presence of cancer similar to our own have been published,^7,8,9,11,12,13^ with very few attempts to externally validate these findings in a further prospective cohort of patients from several centers.

The objective of this multicenter validation study was to establish the diagnostic accuracy of a previously identified set of breath VOCs dysregulated with the presence of OGC in a multicenter setting.

## Methods

Multivariable logistic regression model (stepwise regression) (eMethods 1 in the Supplement) was used to create a 5-VOCs model which were butyric acid, pentanoic acid, hexanoic acid, butanal, and decanal from our previously published data set.^10^ The predictive probabilities generated by this 5-VOC diagnostic model were then used to generate an receiver operating characteristic (ROC) curve, which showed a good diagnostic accuracy with an area under the curve of 0.90 (SD, 0.02).

Based on 50% of patients in the study population having cancer (1 patient with a benign abnormality was recruited to 1 patient with cancer) and maintaining a sensitivity and specificity of 80% for the diagnostic model derived from our previous research, the sample size estimated for the multicenter external validation study was 325 patients; 162 patients with esophageal or gastric cancer and 163 patients with benign conditions or a normal upper gastrointestinal tract.

Breath samples were taken from 3 hospitals (St Mary’s Hospital Imperial College London, University College London Hospital, and The Royal Marsden Hospital) and transported to St Mary’s VOC laboratory for SIFT-MS analysis. The National Health Service (NHS) Health Research Authority (NRES Committee London–Camden and Islington) approved the study, and written informed consent was obtained.

The full protocol for this study was previously published.^14^ The study was reported according to STARD 2015 (Standards for Reporting of Diagnostic Accuracy Studies) guidelines (eMethods 2 in the Supplement).^15^ The trial was registered with the National Health Service, Health Research Authority

Patients 18 years or older with upper gastrointestinal symptoms attending for endoscopy or surgery were eligible. In the cancer cohort only patients with histologically confirmed nonmetastatic esophagogastric adenocarcinoma (stage I-III) were included. All patients in the cancer cohort were sampled when they were neoadjuvant naive.

Patients who had a documented active infection, were unable to provide informed consent, or unable to provide a 500-mL breath sample were excluded. Patients with Barrett esophagus were excluded from the control group (this is a premalignant condition worthy of independent investigation).

### Breath Sampling Methodology

After informed consent was obtained from all patients, we followed the sampling protocol used in our previous clinical studies,^10^ which was informed by our investigations on the influence of breath maneuvers and hospital environment on VOC measurements.^16,17^ Patients fasted for a minimum of 4 hours prior to their breath sample collection. Patients rested in the same area for at least 20 minutes prior to exhaled breath collection and all samples were obtained immediately prior to endoscopy or surgery. Patients were asked to perform a single deep nasal inhalation followed by complete exhalation via their mouth into secure 500-mL steel breath bag (GastroCHECK) via a 1-mL Luer lok syringe (Terumo Europe, Leuven, Belgium). Patients in the cancer and control groups were recruited consecutively. The research team were aware of clinical diagnosis when breath sampling the patients, however the clinical team performing the OGD or surgery were blind to the results of the breath analysis.

For each VOC measurement, the syringe plunger was removed from the 1-mL Luer lok syringe and the steel breath bag was directly connected via the syringe barrel to the sample inlet arm of the SIFT-MS instrument. For the multi-ion monitoring mode, selective VOCs from breath were analyzed for a total of 60 seconds and measured concentrations were averaged over this time for each VOC.

SIFT-MS permits online, real-time VOC quantification.^18,19^ It has been used in the study of VOCs in breath and urine from patients with conditions including cystic fibrosis and bladder cancer.^20,21^ The SIFT-MS instrument allows real-time detection and quantification of VOCs in biological samples such as exhaled breath without sample preparation.^22^ We have previously confirmed the reproducibility of VOCs measurements using SIFT-MS.^23^ A panel of 30 VOCs including the 5 VOCs forming our diagnostic model were analyzed for each breath sample, as previously described.^10^

### Clinical Data

A detailed medical proforma was completed by the consenting clinician or research fellow using information provided by the patient as well as clinical investigations. These data included patient demographics, tumor characteristics, comorbidities, medications, and lifestyle measures. Diagnostic endoscopy and/or operative findings were recorded for each patient.

Histopathologic examination of tissues obtained via endoscopy or from surgically resected specimens was carried out. The reference test was considered positive on OGC histopathologic diagnosis.

SIFT-MS instrument was calibrated daily to 6% water in human exhaled breath. All breath samples were tested using SIFT-MS to ensure that percentage water from the exhaled breath sample in the bag was between 5% and 7%. If this was not the case the sample was discarded because it was likely to be unreliable and representative of bag malfunction. All samples were analyzed within 4 hours of collection. Our methodological studies demonstrated the stability of trace VOCs up to 48 hours from the time of patient sampling when using the GastroCHECK steel breath bag (eMethods 3 in the Supplement). Weekly samples were taken from the ambient room air at the participating hospitals where patients were being breath sampled and also from the laboratory air from where samples were analyzed. This was to ensure that there was no contamination from the ambient room air causing anomalous results, which could represent an important confounding factor (eMethods 4 in the Supplement). Breath sampling methodology was standardized. We performed human factors analysis, which demonstrated several potential sources of error in breath sampling that can affect the results of the analysis. Therefore, all clinicians and researchers participating in this study underwent a thorough credentialing process involving observation of consent, performing breath sampling, and storage of samples prior to participating in the study (eMethods 5 in the Supplement). Threshold of detection of SIFT-MS analysis was defined as 1 part per billion by volume (ppbv), based on previously performed statistical modeling (eMethods 6 in the Supplement).

To confirm the identified VOCs obtained in the exhaled breath using SIFT-MS; we conducted cross-platform validation with Gas chromatography mass spectrometry ([GC-MS] considered the gold standard for compound identification owing to the use of chromatographic separation). Exhaled breath was collected using the same method from 20 patients. The VOC content from each GastroCHECK bag was transferred using an air-sampling pocket pump (SKC 210-1002 series) at 50 mL/min onto inert coated stainless steel Tenax/Carbograph-5TD sorbent tubes (Markes International Ltd, Llantrisant) prior to GC-MS analysis (eMethods 7 in the Supplement).

### Statistical Analysis

Comparison of predicted cancer risk and actual OGD findings or histology from endoscopic biopsies (reference standard test) was then made, and the overall diagnostic accuracy (sensitivity, specificity, and ROC analysis) for this noninvasive diagnostic investigation was determined. A similar ROC analysis was performed based on predicted cancer risk from clinical parameters defined by National Institute for Health and Care Excellence (NICE) criteria.^3^ Potential confounding factors across the study groups were evaluated by employing the Kruskal-Wallis test for continuous variables and χ^2^ test for discrete variables. Linear regression models were used to assess any influence of patient demographic factors, or medications, on VOC concentrations measured. *P* < .05 was used to assign statistical significance. All statistical analysis was performed using the statistical software SPSS (version 22, IBM).

## Results

Only 1 invited patient declined to participate in the study with a patient acceptability rate of 99.7% to undertake and complete the test. No adverse events were observed during breath sampling.

### Patient Demographics and Tumor Factors

After necessary exclusions owing to sample quality, defined as inadequate percentage of water (n = 20), 335 patients in total were included; 172 patients in the control group and 163 patients with esophageal or gastric cancer. In the control group, 89 (51.7%) patients had a normal upper gastrointestinal tract on endoscopy or only the presence of a hiatal hernia. The most common diagnoses among the remaining participants in the control group were esophagitis, gastritis, or duodenitis with or without erosions in 59 (34.3%) patients, followed by the presence of benign gastric polyps in 12 (7.0%) patients and achalasia or esophageal stricture in 11 (6.4%) patients.

In the cancer group there were significant increases in patient age, proportion of male and white patients, ex-smokers, ASA grade 3, and hypertensive patients, with a reduced proportion of patients with liver impairment (Table 1). There were also significant increases in the use of statin, β-blocker, and ACE-inhibitor medications in the cancer group (Table 1). Dysphagia, vomiting, and gastrointestinal bleeding were increased, and abdominal pain reduced, as presenting symptoms in the cancer group (eMethods 8 in the Supplement). Furthermore, the breakdown of the cancer-specific factors including stage and tumor location is provided in Table 2 with 72 (44.2%) of tumors being gastric in origin, 123 (69.3%) being T3 or T4, and 106 (65%) being nodal positive.

**Table 1.** Comparison of Demographic Factors and Medication Between Cancer and Control Patients

Variable	Control Group, No. (%)	Cancer Group, No. (%)	*P* Value
Age, median (IQR)	55 (41–69)	68 (60–75)	<.001
Sex			
Female	91 (52.6)	29 (17.8)	<.001
Male	81 (47.4)	134 (82.2)	
White	88 (51.5)	114 (69.9)	.001
Smoking history			
Current	31 (18.1)	22 (13.5)	<.001
Ex smoker	40 (23.4)	72 (44.2)	
Alcohol history			
Current	77 (45.0)	87 (53.4)	.30
Ex alcohol user	19 (11.1)	17 (10.4)	
ASA grade			
1	72 (42.1)	41 (25.2)	.001
2	91 (53.2)	101 (62.0)	
3	8 (4.7)	21 (12.9)	
Comorbidities			
Diabetes	28 (16.4)	26 (16.0)	.92
Renal impairment	8 (4.7)	3 (1.8)	.15
Chronic obstructive pulmonary disease	10 (5.8)	7 (4.3)	.52
Ischaemic heart disease	19 (11.1)	20 (12.3)	.74
Liver impairment	16 (9.4)	1 (0.6)	<.001
Hypertension	45 (26.3)	62 (38.0)	.02
Asthma	18 (10.5)	19 (11.7)	.74
Medication			
Proton pump inhibitor	83 (48.5)	93 (57.1)	.12
Statin	35 (20.5)	56 (34.4)	.004
β-Blocker	12 (7.0)	27 (16.6)	.007
ACE inhibitor	13 (7.6)	37 (22.7)	<.001
Amlodipine	17 (9.9)	17 (10.4)	.88
Aspirin	13 (7.6)	14 (8.6)	.74
Clopidogrel	7 (4.1)	5 (3.1)	.62
Metformin	20 (11.7)	17 (10.4)	.71
Diuretic	4 (2.3)	7 (4.3)	.31

Abbreviations: ACE, angiotensin converting enzyme; ASA, American Society of Anesthesiologists.

**Table 2.** Description of Cancer-Specific Factors

Tumor-Related Factor	Patients, No. (%)
Tumor location	
Gastric	72 (44.2)
Gastroesophageal junction	36 (22.1)
Oesophageal	55 (33.7)
Clinical T stage	
1	18 (11.0)
2	32 (19.6)
3	61 (37.4)
4	52 (31.9)
Clinical N stage	
0	57 (35.0)
1	58 (35.6)
2	22 (13.5)
3	26 (16.0)

### Cross-Platform GC-MS Validation

In conjunction with SIFT-MS analysis, TD-GC-MS analysis was applied to breath from the same cohort of patients (n = 20) to cross-validate the identity of measured VOCs. eMethods 9 in the Supplement summarizes the VOC identification by GC-MS via mass spectrum (MS) matching of detected compounds to the commercial NIST library, as well as their calculated retention indices (RI) to those of authentic chemical standards undergone separation on the ZB-624 column. Overall, the presence of 30 VOCs was confirmed except for pentanol owing to its limited level of signal-to-noise ratio (SNR<5) among the analyzed samples.

### SIFT-MS Analysis and VOC-Based OGC Diagnosis

The concentration of butyric acid, hexanoic acid, butanal, and decanal showed significant differences between the cancer and control groups (eMethods 10 in the Supplement). Five VOCs were then taken forward to form a risk-prediction model for the diagnosis of esophagogastric cancer, and included in a multivariable logistic regression analysis with cancer diagnosis as the dependent variable (eMethods 10 in the Supplement). To ensure these factors were not associated with a confounding demographic variable or presenting symptom that differed between the comparison groups, linear regression models were performed for each of the 5 VOCs (eMethods 10 in the Supplement). There were no significant differences in the concentration of these 5 VOCs between patients with esophageal or gastric cancer (eMethods 10 in the Supplement).

The predictive probabilities generated by this 5-VOC diagnostic model were then used to generate an ROC curve, which showed a good diagnostic accuracy with an area under the curve of 0.85 (SD, 0.02) (Figure). This translated to a sensitivity of 80% and specificity of 81% for the diagnosis of esophagogastric cancer. This compared with the diagnostic accuracy generated by a clinical parameters test based on NICE guidelines for endoscopy referral,^3^ which had an area under the curve of 0.73 (SD, 0.03), sensitivity of 59%, and specificity of 81% (eMethods 11 in the Supplement).

**Figure.**
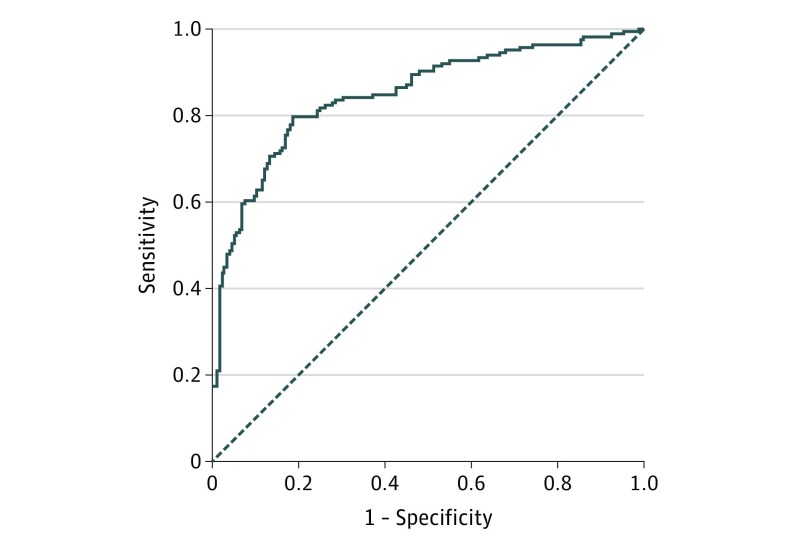
ROC Curve for the 5-VOC Breath Model in the Diagnosis of Esophagogastric Cancer in the Multicenter Clinical Trial^a^ Abbreviations: ROC, receiver operating characteristic curve; VOC, volatile organic compounds. ^a^Area under the curve of 0.85 (SD, 0.02).

## Discussion

This multicenter study demonstrated a sensitivity of 80% and specificity of 81% of a single breath test in the diagnosis of esophagogastric cancer, thus validating the 5-VOC breath model. All patients with cancer included in the study were receiving a curative treatment pathway, highlighting the potential value of the test in detecting operable disease and the potential impact on survival. The sensitivity of 80% compares favorably to the existing technologies such as fecal occult blood test (sensitivity ranging from 30%-70%) for colorectal cancer,^24^ and more specifically to upper gastrointestinal disease, the cytosponge (sensitivity 73%) for Barrett esophagus.^25^ An important finding with both these technologies was an increase in sensitivity associated with multiple episodes of testing, which could be an important area for further research of breath testing.

At present the NICE guidance for endoscopy referral is based on age threshold and symptom criteria. Patients aged over 55 years with dyspepsia, or those of any age with alarm-type symptoms are considered eligible for direct referral for endoscopy and assessment of the upper gastrointestinal tract.^3^ Despite these guidelines a huge degree of variability remains in referral patterns for endoscopy.^4^ The breath test for esophagogastric cancer aims to provide clinicians with an objective assessment of need for endoscopic referral. Given the association of all 5 VOCs with esophagogastric cancer, this may in the future allow for calculation of stratified risk for individual patients, which would require an independent large-scale study to fully validate. The consensus of key stakeholders in a decision workshop was to locate breath testing in primary care to triage patients with nonspecific symptoms to have endoscopy based on risk of OGC (eMethods 12 in Supplement 2). This view has been endorsed by our recent finding that the diagnostic model for OGC is different from that for colorectal cancer,^26^ providing the concept for a single breath test for multiple gastrointestinal cancers. If a clinician is presented with a patient with gastrointestinal symptoms that do not prompt referral based on NICE criteria, he/she would not need to watch and wait to see if symptoms worsen but could offer the exhaled breath test immediately. The clinician would order a breath test in much the same way as routine blood tests. A nurse can perform the test and send breath samples to a regional laboratory for analysis. A positive result would warrant immediate referral for endoscopy. A negative test would permit the clinician to reassure the patient and offer retesting if symptoms persist. Because endoscopy is an expensive investigation,^1^ with a low diagnostic yield of 2% to 5%,^2^ a triage breath test prior to endoscopy could substantially reduce the number of negative endoscopies and increase the cancer yield making the diagnostic pathway more effective with improved patient experience. Avoiding unnecessary investigations would also free up resources in the NHS. Concerns regarding clinical application of breath sampling and transport have led to the development of thermal desorption tubes, which allow breath samples to be stored for up to 1.5 months and transported between sites.^27,28^ These tubes can be used multiple times after cleaning and potentially for multiple diseases using the same analytical platform, which may serve to further lower the cost of a breath test.

The mechanism of production of these VOCs in the cancer state may involve changes at a genetic and cellular level causing metabolic alterations in enzymatic pathways. Aldehydes have generated much research interest given their link as a possible carcinogen and also their elevation in other types of cancers.^28^ Genetic dysregulation of aldehyde metabolism is present in patients with esophageal cancer.^29,30^ Lipid peroxidation flux may provide a link between inflammation, aldehydes, and cancer.^12,31^ Gastric microbiome associated with cancer may also be a contributing factor to the production of VOCs, yet to be defined.^32,33^

### Limitations

There are limitations associated with this study that must be considered in the interpretation of the findings. Although demographic data were collected and regressed for in the analysis, there may have been other unmeasured confounding variables that could have influenced the changes in VOCs observed. Also, the reference standard test was histopathologically proven tissue diagnosis through endoscopy or from surgically resected specimen, although gastric and esophageal cancers can be missed in up to 8% of diagnostic endoscopies,^34,35^ however endoscopy remains the best diagnostic test currently available. Furthermore, most patients presented in the current study have T3 esophagogastric cancer, in line with disease patterns in the UK. Therefore the diagnostic accuracy of the test to identify early stage (T1) cancer remains undetermined by the current study. It must be acknowledged that given a current sensitivity of 80% there is still potential for further refinement of exhaled breath testing and thereby improvements in cancer detection rates; a successful evolution observed in other triage investigations such as stool DNA testing for colorectal cancer.^36,37^ Further investigations are also needed to examine the sensitivity of breath analysis on multiple testing samples in patients who initially have a negative result.

## Conclusion

This validation study showed a sensitivity of 80% and a specificity of 81% for the breath test to diagnose esophagogastric cancer. The next stage is a large-scale diagnostic accuracy study among the primary care population where the test is intended to be employed.

## Notes

Supplement. eMethods 1: VOC breath model refinement eMethods 2: STARD 2015 list eMethods 3: Optimisation of Bag materials eMethods 4: Effect of ambient room air upon analysis of trace VOCs eMethods 5: Human factor analysis of breath bag sampling eMethods 6: Detection limit of SIFT-MS identified as 1ppbv eMethods 7: GC-MS analysis eMethods 8: Comparison of presenting symptom between cancer and control patients eMethods 9: GC-MS cross platform validation eMethods 10: Comparison of VOCs concentrations between cancer and controls and regression for confounding variables eMethods 11: Diagnostic accuracy of test based upon clinical parameters for NICE guidelines for endoscopy referral eMethods 12: Decision conferencing – patient pathway
