# Supplementary material for: Assessment of a Noninvasive Exhaled Breath Test for the Diagnosis of Oesophagogastric Cancer
Source: JAMA Oncol. 2018 May 17;4(7):970–6. doi: 10.1001/jamaoncol.2018.0991 (PMC6145735; doi:10.1001/jamaoncol.2018.0991)
Supplement: Supplement. — eMethods 1: VOC breath model refinement eMethods 2: STARD 2015 list eMethods 3: Optimisation of Bag materials eMethods 4: Effect of ambient room air upon analysis of trace VOCs eMethods 5: Human factor analysis of breath bag sampling eMethods 6: Detection limit of SIFT-MS identified as 1ppbv eMethods 7: GC-MS analysis eMethods 8: Comparison of presenting symptom between cancer and control patients eMethods 9: GC-MS cross platform validation eMethods 10: Comparison of VOCs concentrations between cancer and controls and regression for confounding variables eMethods 11: Diagnostic accuracy of test based upon clinical parameters for NICE guidelines for endoscopy referral eMethods 12: Decision conferencing – patient pathway [file jamaoncol-4-970-s001.pdf]

Supplementary Online Content

Markar SR, Wiggins T, Antonowicz S, et al. Assessment of a noninvasive exhaled breath test for the diagnosis of oesophagogastric cancer [published online May 17, 2018]. *JAMA Oncol*.
doi:10.1001/jamaoncol.2018.0991

eMethods 1: VOC breath model refinement
eMethods 2: STARD 2015 list
eMethods 3: Optimisation of Bag materials
eMethods 4: Effect of ambient room air upon analysis of trace VOCs
eMethods 5: Human factor analysis of breath bag sampling
eMethods 6: Detection limit of SIFT-MS identified as 1ppbv
eMethods 7: GC-MS analysis
eMethods 8: Comparison of presenting symptom between cancer and control patients
eMethods 9: GC-MS cross platform validation
eMethods 10: Comparison of VOCs concentrations between cancer and controls and regression for confounding variables
eMethods 11: Diagnostic accuracy of test based upon clinical parameters for NICE guidelines for endoscopy referral
eMethods 12: Decision conferencing – patient pathway

This supplementary material has been provided by the authors to give readers additional information about their work.

© 2018 Markar SR, et al. *JAMA Oncology.*

eMethods 1: VOC breath model refinement

Methods

SIFT-MS data from the previous publication by Kumar et al, included 210 patients, 81 with oesophageal or gastric adenocarcinoma and 129 control patients. Given the challenges around separation of phenol and the difficulties associated with transport of phenolic based VOCs, it was considered that in a multi-centre study the analysis of phenolic based VOCs would be unreliable using SIFT-MS, and therefore they were excluded from the diagnostic model. This left ten VOCs that were taken forward to generate a new diagnostic model from this obtained dataset (e*Table 1).* The concentrations of all VOCs from these data were compared using univariate statistics, Mann-Whitney-U test, across cancer and non-cancer groups. These ten VOCs were taken forward into a multivariable logistic regression model with the dependent variable being the presence of oesophago-gastric cancer. Significant VOCs from this multivariable analysis were defined by statistical significance of P<0.05, and these were taken forward to another multivariable logistic regression model (stepwise regression). Results are presented as odds ratios and 95% confidence intervals. To construct the Receiver Operating Characteristic (ROC) curves, cancer status was used as the dependent variable and the sum concentrations of significant VOCs from the multivariable logistic regression model were used as the independent variable. All statistical analysis was performed using the statistical software SPSS (version 22).

eTable 1: Ten VOCs taken forward for further investigation

VOC	Molecular formula	Precusor ions	m/z	Characteristic product ion
Butyric acid	$C_4H_8O_2$	H_30^+	89	$C_4H_8O_2H^+$
Pentanoic acid	$C_5H_{10}O_2$	H_30^+	103	$C_5H_{10}O_2H^+$
Hexanoic acid	$C_6H_{12}O_2$	H_30^+	117, 135	$C_6H_{12}O_2H^+$ $C_6H_{12}O_2H^+(H_20)$
Butanal	C_3H_7CHO	NO^+	71	$C_4H_7O^+$
Pentanal	C_4H_9CHO	NO^+	85	$C_5H_9O^+$
Hexanal	$C_5H_{11}CHO$	NO^+	99	$C_6H_{11}O^+$
Heptanal	$C_6H_{13}CHO$	NO^+	113	$C_7H_{13}O^+$
Octanal	$C_7H_{15}CHO$	NO^+	127	$C_8H_{15}O^+$
Nonanal	$C_8H_{17}CHO$	NO^+	141	$C_9H_{17}O^+$
Decanal	$C_9H_{19}CHO$	NO^+	155	$C_{10}H_{19}O^+$

Results

Univariate comparison performed for all VOCs measured in the previous dataset demonstrated significant for 15 VOCs (eFigure 1). All ten VOCs described in etable 2 were significantly dysregulated in the cancer state.

eFigure 1: Graphically illustrating changes in all VOCs between study groups. Positive deflection indicated an upregulation in the cancer group and a negative deflection indicated a downregulation in the cancer group relative to the non-cancer group.

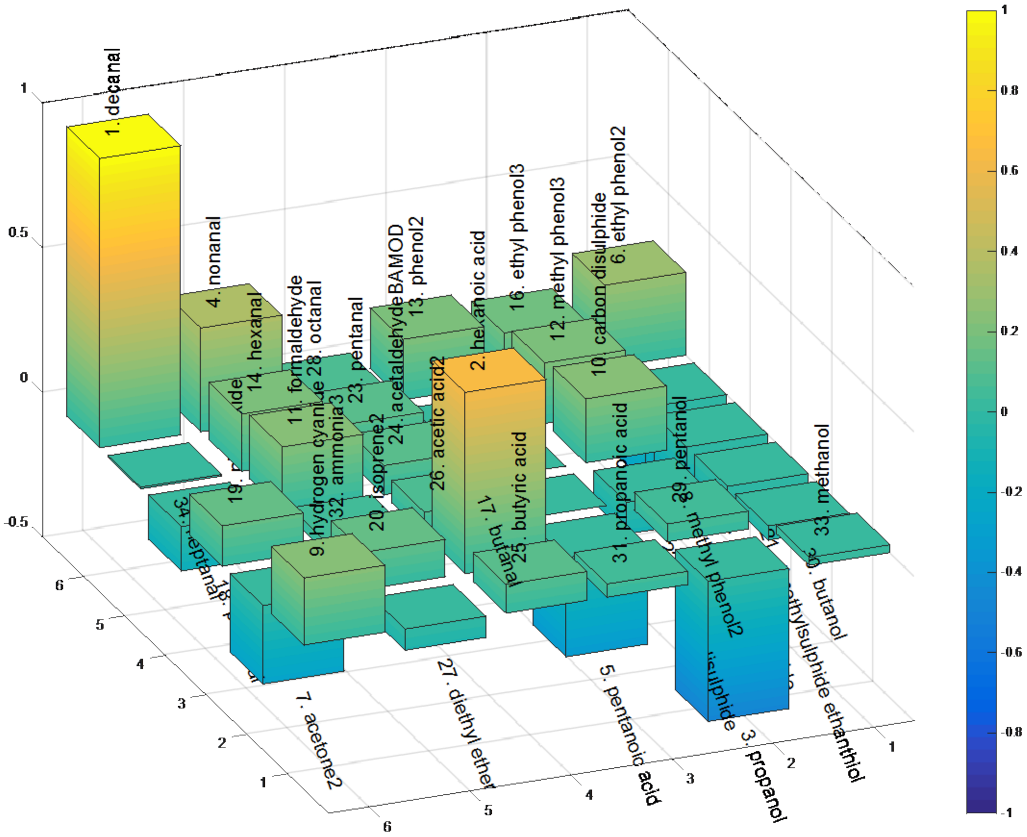

The ten VOCs described in *etable 1* were then taken forward to a multivariable analysis, with the results showing significant associations between the presence of cancer and five VOCs which were butyric acid, pentanoic acid, hexanoic acid, butanal and decanal (*etable 3*).

eTable 2: Results of multivariable analysis with oesophago-gastric cancer as the dependent variable and then ten VOCs described previously as independent variables

VOC	Odds ratio	95% confidence interval	P value
Butyric acid	1.02	1.00 – 1.03	0.034
Pentanoic acid	0.94	0.90 – 0.97	0.001
Hexanoic acid	1.14	1.07 – 1.23	<0.001
Butanal	0.81	0.68 – 0.96	0.014
Pentanal	1.02	0.83 – 1.25	0.846
Hexanal	0.99	0.90 – 1.09	0.804
Heptanal	0.89	0.72 – 1.11	0.308
Octanal	0.94	0.77 – 1.14	0.516
Nonanal	1.09	0.92 – 1.29	0.318
Decanal	1.44	1.25 – 1.66	<0.001

© 2018 Markar SR, et al. *JAMA Oncology*.

These five VOCs (butyric acid, pentanoic acid, hexanoic acid, butanal and decanal) were then taken forward to form a separate diagnostic model with multivariate analysis.

eTable 3: Results of multivariable analysis with oesophago-gastric cancer as the dependent variable and five VOCs described previously as independent variables

VOC	Odds ratio	95% confidence interval	P value
Butyric acid	1.02	1.00 – 1.03	0.034
Pentanoic acid	0.94	0.90 – 0.97	0.001
Hexanoic acid	1.14	1.07 – 1.22	<0.001
Butanal	0.79	0.68 – 0.93	0.014
Decanal	1.42	1.25 – 1.60	<0.001

The predictive probabilities generated by this five-VOC diagnostic model was then used to generate a ROC curve, which showed a good diagnostic accuracy with an area under the curve of 0.90 +/- 0.02. This translates to a sensitivity of 84% and specificity of 88% for the diagnosis of oesophago-gastric cancer.

eFigure 2: ROC curve for the 5 VOC breath model in the diagnosis of oesophago-gastric cancer

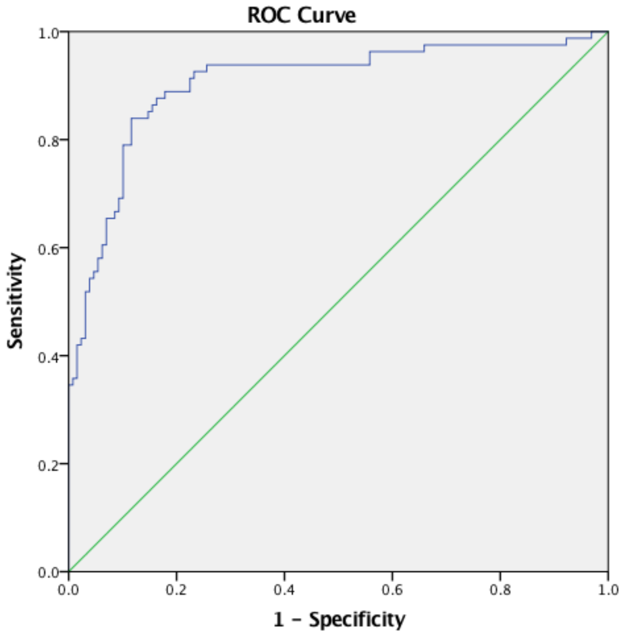

© 2018 Markar SR, et al. *JAMA Oncology*.

eMethods 2: STARD 2015 list

eTable 4

Section and topic	Number	Item	Present study reported (yes or no)
**Title or abstract**	1	Identification as a study of diagnostic accuracy using at least one measure of accuracy (such as sensitivity, specificity, predictive values, or AUC)	Yes
**Abstract**	2	Structured summary of study design, methods, results, and conclusions	Yes
**Introduction**	3	Scientific and clinical background, including the intended use and clinical role of the index test	Yes
	4	Study objectives and hypotheses	Yes
**Methods**			
Study design	5	Whether data collection was planned before the index test and reference standard were performed (prospective study) or after (retrospective study)	Yes
Participants	6	Eligibility criteria	Yes
	7	On basis potentially eligible participants were identified (such as symptoms, results from previous tests, inclusion in registry)	Yes
	8	Where and when potentially eligible participants were identified (setting, location, and dates)	Yes
	9	Whether participants formed a consecutive, random, or convenience series	Yes
	10a	Index test, in sufficient detail to allow replication	Yes
	10b	Reference standard, in sufficient detail to allow replication	Yes
	11	Rationale for choosing the reference standard (if alternative exists)	Yes
	12a	Definition of and rationale for test positivity cut-offs or result categories of the index test, distinguishing pre-specified from exploratory	Yes
	12b	Definition of and rationale for test positivity cut-offs or result categories of the reference standard, distinguishing pre-specified from exploratory	Yes
	13a	Whether clinical information and reference standard results were available to the performers or readers of the index test.	Yes
	13b	Whether clinical information and index test results were available to the assessors of the reference standard	Yes
Analysis	14	Methods for estimating or comparing measures of diagnostic accuracy	Yes
	15	How indeterminate index test or reference standard results were handled	Yes

© 2018 Markar SR, et al. *JAMA Oncology*.

	16	How missing data on the index test and reference standard were handled	Yes
	17	Any analyses of variability in diagnostic accuracy, distinguishing pre-specified from exploratory	Yes
	18	Intended sample size and how it was determined	Yes
**Results**			
Participants	19	Flow of participants, using a diagram	No but described in text
	20	Baseline demographic and clinical characteristics of participants	Yes
	21a	Distribution of severity of disease in those with the target condition	Yes
	21b	Distribution of alternative diagnoses in those without the target condition	Yes
	22	Time interval and any clinical interventions between index test and reference standard	Yes
Test results	23	Cross tabulation of the index test results (or their distribution) by the results of the reference standard	No
	24	Estimates of diagnostic accuracy and their precision (such as 95% confidence intervals)	Yes
	25	Any adverse events from performing the index test or the reference standard	Yes
**Discussion**	26	Study limitations, including sources or potential bias, statistical uncertainty and generalisability	Yes
	27	Implications for practice, including the intended use and clinical role of the index test	Yes
**Other information**	28	Registration number and name of the registry	Yes
	29	Where the full study protocol can be accessed	Yes
	30	Sources of funding and other support; role of funders	Yes

© 2018 Markar SR, et al. *JAMA Oncology*.

eMethods 3: Optimisation of Bag materials

We conducted an experiment to optimise the bag materials to minimise losses of trace VOCs as part of a multi-centre investigation.

Liquid Calibration Unit (LCU)

The loading of the bags with known amounts of linear aliphatic aldehydes was carried out with the aid of a liquid calibration device (s-LCU from Ionicon Analytik, GmbH - Innsbruck, Austria). The calibration mixture was generated by injecting a mixture of aldehydes (propanal to heptanal, at 1·0-1·7 mg/L in water) at a flow of 50 ml/min into a heated (100°C) chamber. Upon injection, the liquid encountered a gas stream, flowing at 1,000 ml/min; this allowed for rapid evaporation of the analytes, due to the generation of micro-droplets. Knowing the starting concentration of the single aldehydes and supposing the evaporation of the liquid to be instantaneous and quantitative, this should generate C3-C7 linear aliphatic aldehydes in the low-parts-per billion volume (ppbv) range. This assumption was experimentally verified by connecting the LCU device to the SIFT-MS. C3-C6 aldehydes showed a good agreement between expected and measured values, with concentrations in the ±20% range with respect to theoretical values. In the case of heptanal, the measured concentration was repeatable, but considerably lower than the theoretical one. This was probably due to poor evaporation efficiency, also observed for higher boiling point aldehydes, which were evaluated in a preliminary experiment (C8 to C10). The relative humidity of the obtained calibration mixture was 6·2%, and therefore similar to that occurring in breath. The gas stream injection was achieved by means of pressurised gas (synthetic air, BOC gases - Guildford, UK), passed through a scrubber (Supelco - Bellefonte, PA) and connected to the LCU. The calibration mixture was conveyed to the SIFT-MS by means of a short (10 cm) section of PEEK tubing. For the multi-ion monitoring mode, selective VOCs (trace aldehydes) from breath were analysed for a total of 60s and measured concentrations were averaged over this time for each VOC.

Bag materials under investigation were Nalophan (Kalle Ltd, Germany), Tedlar (Sigma Aldrich Ltd., Poole - UK), and Steel (Gastrocheck-Bag-XL-Bedfont Scientific Limited). Bags were stored at room temperature, and were sampled at 0, 24, 48, and 72 hours. Three bags were sampled at each time point with the median and range presented for analysis. Kruksall-Wallis test was utilised to compare the concentration of the trace VOCs at different time points, with a P value of 0.05 taken to indicate statistical significance.

Comparison of the three bag types showed variable performance in the ability to retain water and trace aldehydes over the up to 72-hour study period. When stored in Nalophan for 72 hours, there were significant reductions in water (57·1%), propanal (40·4%), butanal (48·7%) and hexanal (55·2%). Tedlar performed well for most aldehydes, however again there were significant reductions in water (47·9%) and heptanal (54·7%). Steel performed well in the retention of most aldehydes with the exception of pentanal, which showed a 73·9% reduction during the 72-hour study period (e*Table 5 and eFigure 3*).

© 2018 Markar SR, et al. *JAMA Oncology*.

eTable 5: Changes in C3 – C7 aldehyde concentration when stored in different bag materials over time.

TIME (Hours)	0 hr (ppbv (median (range))	24 hrs (ppbv (median (range))	48 hrs (ppbv (median (range))	72 hrs (ppbv (median (range))	P value
**Nalophan**					
**Water**	41231894 (37981654 – 42002213)	28213875 (26936224 – 39068779)	181919133 (18047376 – 20314115)	17680424 (17519346 – 18772673)	0·023
**Propanal**	31·9 (27·5 – 32)	32·6 (27·6 – 33·4)	20·9 (15 – 22·8)	19 (16·3 – 19·6)	0·033
**Butanal**	19·2 (18·7 – 26·2)	17·5 (13·2 – 17·8)	10·8 (8·9 – 11·5)	9 (8·5 – 12·9)	0·025
**Pentanal**	18·9 (11·5 – 21·7)	24·5 (10·8 – 76·7)	11·4 (6·1 – 12)	9·7 (8·3 – 13·7)	0·223
**Hexanal**	14·3 (10·8 – 16·5)	10·8 (10·7 – 14)	5·7 (5·3 – 10·4)	6·4 (5·4 – 8·5)	0·032
**Heptanal**	6·3 (1·8 – 6·5)	2·8 (2 – 3·9)	2·6 (1·5 – 3·9)	3·7 (2 – 4·8)	0·680
**Tedlar**					
**Water**	33467929 (23383793 – 34056315)	17374156 (16719331 – 17855471)	19075033 (18366469 – 21137799)	17448601 (16681339 – 17847354)	0·025
**Propanal**	35 (33·6 – 40·7)	37·7 (23·8 – 47·6)	32·3 (32·1 – 33·6)	34 (32·8 – 36·4)	0·384
**Butanal**	20·7 (19·7 – 21·4)	19·6 (18 – 22·9)	16·9 (15·4 – 17·4)	18·5 (17·2 – 21(2)	0·094
**Pentanal**	24·1 (21·8 – 47·6)	33·4 (24·6 – 33·6)	15·5 (14·3 – 25)	18·1 (17·9 – 21)	0·113
**Hexanal**	18·2 (16·5 – 18·9)	15·7 (14 – 17·2)	11·6 (11·3 – 14·9)	14 (10·6 – 15·4)	0·061
**Heptanal**	14·8 (13·1 – 15·1)	7 (5 – 8·8)	8·6 (7·3 – 9)	6·7 (4·9 – 7·3)	0·043
**Steel**					
**Water**	46275051	47000803	40455301	41621443	0·579

© 2018 Markar SR, et al. *JAMA Oncology*.

	(44274984 – 47000803)	(42398303 – 50310651)	(39658181 – 48245388)	(39707797 – 48679322)	
**Propanal**	33·4 (33·2 – 37·6)	41·9 (37·6 – 42·2)	35 (33·8 – 39·1)	36·1 (34·1 – 36·8)	0·134
**Butanal**	20·1 (20 – 22·9)	13·2 (10·5 – 22·9)	10·1 (8·9 – 14)	10·2 (8·3 – 13)	0·091
**Pentanal**	20·7 (17 – 27·4)	12·6 (9·8 – 27·4)	7·1 (5·6 – 10·4)	5·4 (3·9 – 7·9)	0·042
**Hexanal**	8·1 (7·8 – 11·4)	6·1 (4·4 – 11·4)	4·6 (4 – 4·8)	3·9 (3·1 – 5·7)	0·085
**Heptanal**	3·1 (2·4 – 3·2)	3·2 (1·6 – 6·3)	7 (2·5 – 9·1)	2·5 (1·4 – 3·3)	0·47

© 2018 Markar SR, et al. *JAMA Oncology*.

eFigure 3: Illustrating losses over time of C3 – C7 aldehydes when stored in different bag materials

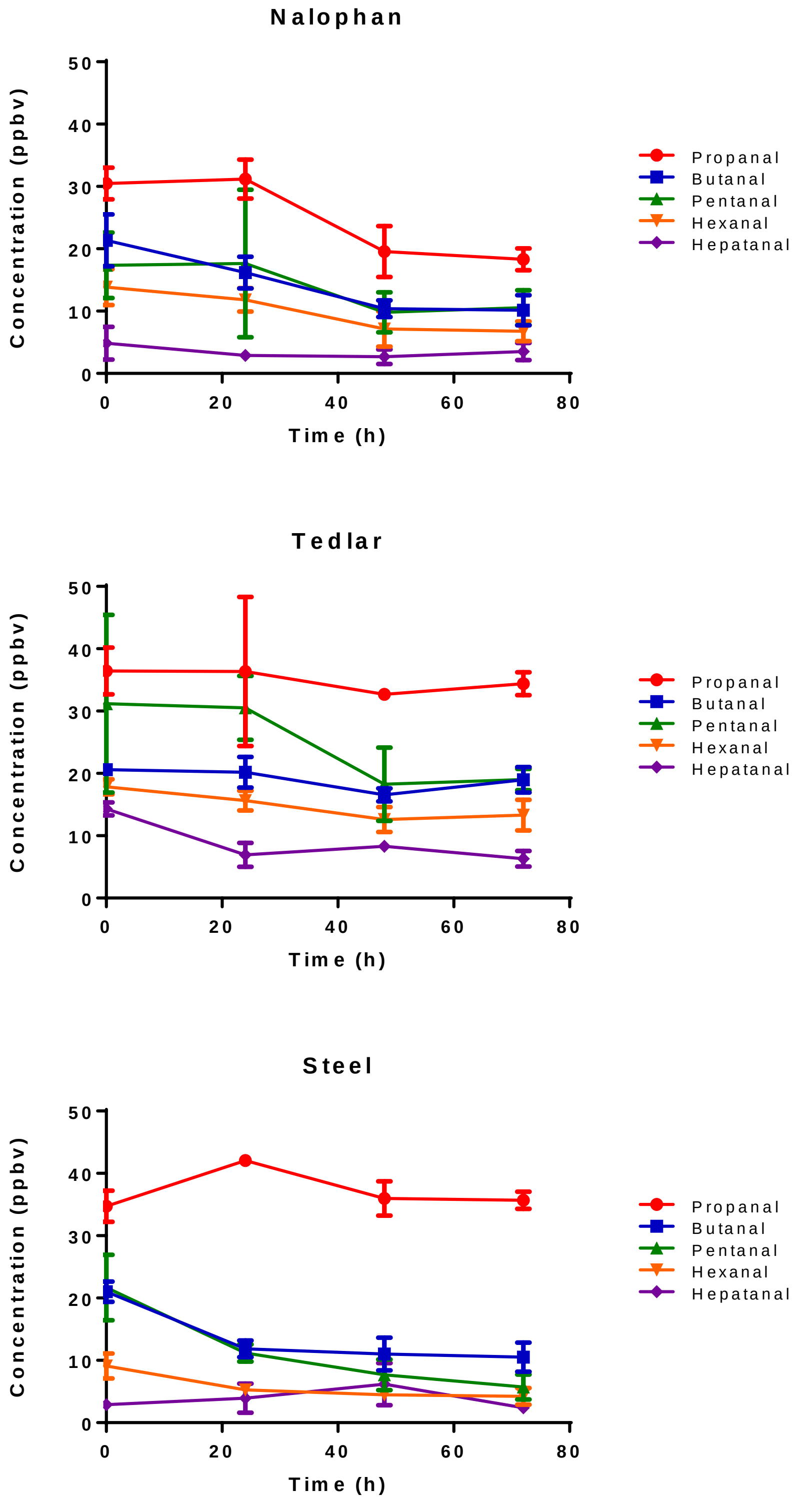

The results of this study demonstrate that there is loss of trace VOCs from breath bags that impair the interpretation of multi-centre breath studies that involve long periods of sample transport and storage. For the purpose of our investigation steel breath bags appear to have the best performance in reducing loss of trace

© 2018 Markar SR, et al. *JAMA Oncology*.

aldehydes. However, the results of the study do highlight the need for minimising storage time and facilitating early SIFT-MS analysis. Therefore we amended our protocol in response to this study so that all breath samples were stored in steel breath bags and analysed within 8 hours of being taken from the patient.

© 2018 Markar SR, et al. *JAMA Oncology*.

eMethods 4: Effect of ambient room air upon analysis of trace VOCs

The primary objective of this study was to examine the variation in the levels of traces VOCs from the ambient air in different clinical environments where patients are commonly sampled. The secondary objective of this study was to evaluate the intra- and inter-day variability in the levels of VOCs in these four locations.
Room air samples were collected in breath bags using a room air pump. Room air samples were on 5 separate days over a 1-month period in the morning and afternoon from 3 hospital environments (outpatient clinic, endoscopy and theatre waiting rooms) and the laboratory.
For each VOC measurement, the syringe plunger was removed from the 1ml Luer lok syringe and the breath bag was directly connected via the syringe barrel to the sample inlet arm of the SIFT-MS instrument. For the multi-ion monitoring mode, selective VOCs from ambient air were analysed for a total of 60s and measured concentrations were averaged over this time for each VOC.
The only significant variation in room air VOCs between rooms was seen for butenal, acrolein, butanol, pentanol, butyric acid, putreisceine, methanol33 and isoprene. Importantly there was no significant variation between hospital environments seen in all VOCs included in the oesophago-gastric cancer prediction model previously generated by Kumar et al [8].

eTable 6: Variation in the concentration of fatty acids and phenols observed in the room air from different hospital environments and the laboratory. All values presented as median (range) in ppbv

VOC	Laboratory	Clinic	Endoscopy	Theatre	P value
Butyric acid	4.8<br>(2 – 5.6)	20.2<br>(10.7 – 29.5)	21.9<br>(18.5 – 33.9)	17.1<br>(7.2 – 38.5)	0.034
Pentanoic acid	0.6<br>(0.3 – 3.7)	1.4<br>(0.6 – 7.2)	1.3<br>(0.3 – 6.7)	0.2<br>(0 – 2.8)	0.292
Hexanoic acid	1.5<br>(0.3 – 4.4)	1.1<br>(0 – 3.6)	1.0<br>(0 – 2.9)	1.4<br>(0.4 – 4)	0.848
Phenol	1.8<br>(0.6 – 14.2)	3.1<br>(0 – 14)	1.5<br>(0 – 17)	4.3<br>(1 – 9.5)	0.982
Methyl-phenol	2.2<br>(0 – 9)	1.9<br>(0.6 – 4.4)	0.9<br>(0 – 9.1)	6.7<br>(0.7 – 27.3)	0.572
Ethyl-phenol	1.4<br>(0 – 16.6)	2.1<br>(0 – 18.3)	2.5<br>(0 – 23.7)	6.2<br>(0.5 – 16.2)	0.907

© 2018 Markar SR, et al. *JAMA Oncology*.

eTable 7: Variation in the concentration of aldehydes observed in the room air from different hospital environments and the laboratory. All values presented as median (range) in ppbv

VOC	Laboratory	Clinic	Endoscopy	Theatre	P value
Propanal	48.6 (20.6 – 50.2)	11.6 (9.8 – 12.9)	19 (9 – 29)	14.7 (6.9 – 23)	0.080
Butanal	1 (0 – 3.5)	1.5 (0.6 – 2)	1.7 (0.3 – 3.2)	1.8 (0.3 – 4.4)	0.862
Pentanal	0.4 (0 – 4.2)	2.9 (2.1 – 4.2)	1.2 (0.8 – 16)	1 (0.3 – 5.5)	0.30
Hexanal	0.4 (0 – 2.1)	3.1 (0.7 – 9.1)	2.2 (0.4 – 10.4)	1.9 (0.3 – 6.5)	0.216
Heptanal	0.7 (0 – 2.4)	0.6 (0 – 1.1)	2.9 (0.8 – 1.7)	1 (0.4 – 3.5)	0.204
Octanal	0.3 (0 – 5.7)	2.7 (1.2 – 8.5)	4.4 (0 – 9.1)	3.4 (0.4 – 9)	0.472
Nonanal	0 (0 – 7.4)	0.2 (0 – 2.9)	0.8 (0.5 – 0.9)	1.9 (0 – 9.1)	0.429
Decanal	0 (0 – 5.8)	0.8 (0 – 4.8)	0.3 (0 – 7.5)	2.9 (0 – 5.8)	0.840

Room air from different clinical environments has previously been shown to vary in terms of more abundant VOCs. The present study identifies minimal variation in trace VOCs associated with oesophago-gastric cancer from previous research. However good scientific practice will remain to sample ambient room air at the time of breath sampling to ensure, that exogenous contribution to the patient breath profile is minimal. Regular ambient room air sampling was therefore included as part of the protocol for all clinical samples taken as part of this research.

© 2018 Markar SR, et al. *JAMA Oncology*.

eMethods 5: Human factor analysis of breath bag sampling

Previous breath research has most commonly involved one or two well-trained researchers taking breath samples from individual patients. Single centre breath studies are of value in establishing pilot research findings, however require validation in larger scale multi-centre studies in order to demonstrate reproducibility of findings. This present study sought to utilise human factor analysis to identify potential sources of error in the breath sampling and analysis process that may lead to errors in sample study and spurious results.

Clinicians and researchers undertaking breath sampling were directly observed or videoed during the first three times they performed breath sampling from patients using the 500mL Steel breath bag (Gastrocheck-Bag-XL-Bedfont Scientific Limited). Human factors and Ergonomic (HFE) analysis was employed to identify potential errors and the consequences of these errors associated with the breath sampling technique. HFE is a multidisciplinary science in which human behavior, capacities, and engineering principles are used to explore why errors occur, and how to reduce the likelihood or preventable harm to individuals, with the specific aim to support human performance and safety.

The observation of 3 clinicians and 2 researchers during the first 3 episodes of breath sampling identified 10 tasks with associated errors and consequences associated with breath bag sampling. From this, a task analysis was developed (*Table S1*) that allows assessment of researchers before permitting them to enroll patients in multi-centre breath studies.

eTable 8: Task analysis of breath sampling with steel breath bags.

TASK	CONSEQUENCE
1. Remove plunger	a. Cannot fill bag<br>b. Time lost
2. Remove blue piece	a. End expiratory volume not sampled<br>b. Time lost
3. Placement of 1 and 2	a. Cannot find 1 and 2<br>b. Time lost<br>c. Cannot seal bag
4. Explanation of breath sampling to patient	a. Patient does not fill bag<br>b. Contamination of sample<br>c. Time lost
5. Give bag to patient	a. Patient does not fill bag<br>b. Contamination of sample
6. a. Complete exhalation by patient; b. Valve opened by operator	a. End expiratory volume not sampled<br>b. Sample lost
7. Close bag with tight seal	a. Leakage of sample<br>b. Contamination of sample
8. Replace plunger	a. Leakage of sample<br>b. Contamination of sample
9. Replace blue piece	a. Leakage of sample<br>b. Contamination of sample

10. Place Breath sample in plastic box	a. Contamination of sample<br>b. Compression of sample<br>c. Leakage of sample

This task analysis was taken forward and used in practice as part of the multi-centre trial to ensure all researchers were adequately trained to take breath samples, and reduced any previously demonstrated variability in performance of breath sampling.

© 2018 Markar SR, et al. *JAMA Oncology*.

eMethods 6: Detecting limit of SIFT-MS identified as 1ppbv

eFigure 4: Pearson Correlation coefficient

Rho_X,Y = corr(X,Y) = cov(X,Y)/(sig_X*sig_Y) = E[(X-mu_X)(Y-mu_Y)]/(sig_X*sig_Y)

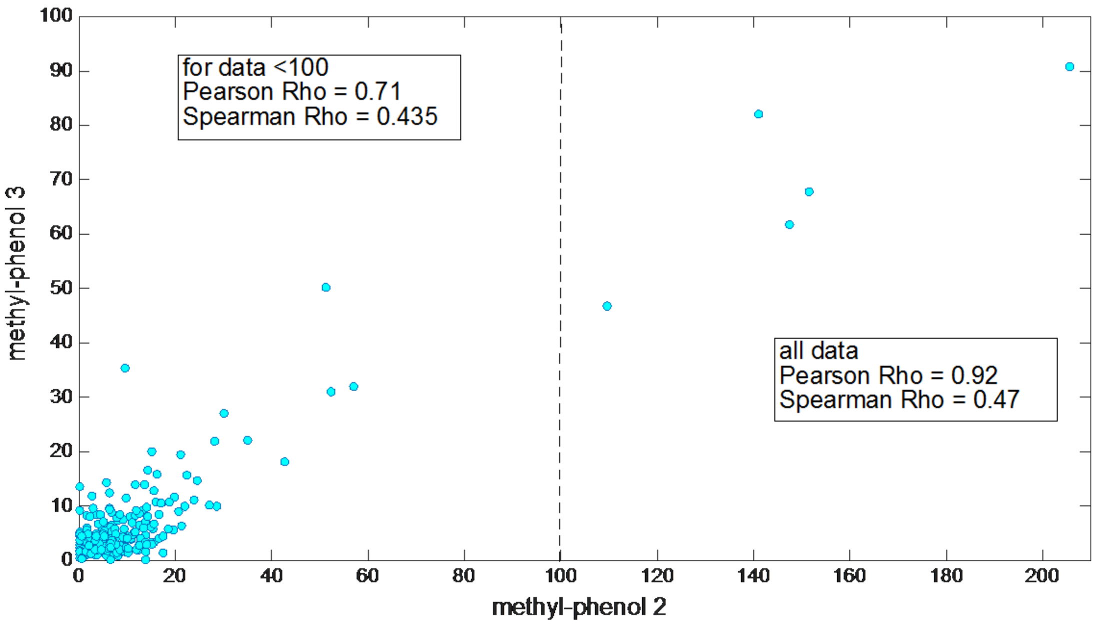

With logarithmic compression of data, question is how to reassign zeros. If zero is 0.1 Rho=0.

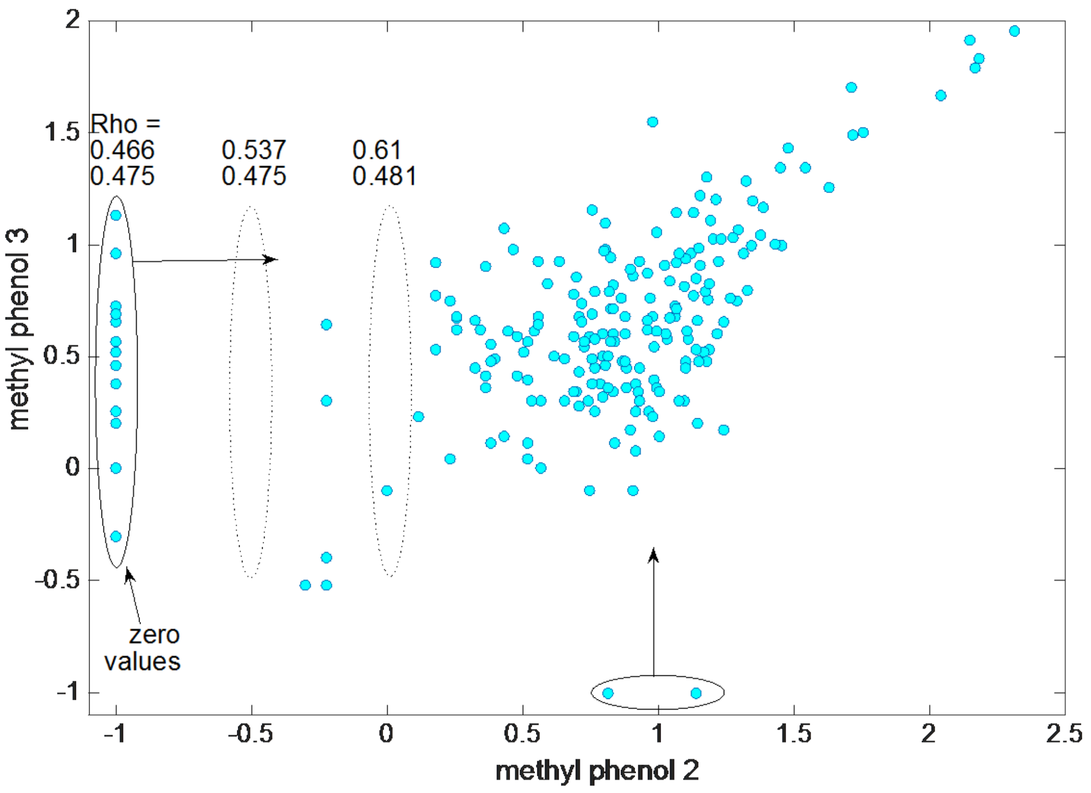

Correlation plot demonstrating a good correlation between methyl phenol measured on NO+ and H3O+, up a value of log 0 or 1ppbv.

© 2018 Markar SR, et al. *JAMA Oncology*.

eMethods 7: GC-MS analysis

An Agilent 7890B GC with 5977A MSD (Agilent Technologies, Cheshire, UK), coupled to a Markes TD-100 thermal desorption unit (TDU) was used. A two-stage thermal desorption program was used at 50 mL/min constant Helium flow rate. In the primary desorption stage, the TD tube sample was dry-purged for 3 min before being heated to 280 °C for 10 min. During secondary desorption stage, VOCs from the cold trap (U-T12ME-2S) were rapidly desorbed from 10 °C to 290 °C at 99 °C/min heating rate and held for 4 min to completely transfer the VOCs onto GC. Flow path from TDU to GC was heated constantly at 140 °C.

VOCs separation was performed on a ZB-624 capillary column (60 m × 0·25 mm ID × 1·40 µm d_f; Phenomenex Inc, Torrance, USA) programmed at 1·0 mL/min Helium carrier. Oven temperature profile was set at 40 °C initially for 4 min, ramp to 100 °C (5 °C/min with 1 min hold), ramp to 110 °C (5 °C/min with 1 min hold), ramp to 200 °C (5 °C/min with 1 min hold), final ramp to 240 °C at 10 °C/min with 4 min hold. The MS transfer line was maintained at 240 °C whilst the EI source was set at 70 eV and 230 °C. MS analyser was set to acquire over the range of 20–250 m/z with data acquisition approximated to 6 scan/sec.

© 2018 Markar SR, et al. JAMA Oncology.

eMethods 8: Comparison of presenting symptom between cancer and control patients.

eTable 9

Symptom	Control group (%)	Cancer group (%)	P value
Heartburn	88 (51·5)	93 (57·1)	0·305
Chest pain	8 (4·7)	11 (6·7)	0·414
Cough	10 (5·8)	4 (2·5)	0·122
Hoarseness	1 (0·6)	0 (0)	0·328
Abdominal pain before eating	49 (28·7)	13 (8·0)	<0·001
Abdominal pain after eating	46 (26·9)	9 (5·5)	<0·001
Dysphagia	33 (19·3)	59 (36·2)	0·001
Odynophagia	4 (2·3)	5 (3·1)	0·681
Gastrointestinal bleeding	6 (3·5)	25 (15·3)	<0·001
Unintentional weight loss	48 (28·1)	54 (33·1)	0·316
Vomiting	19 (11·1)	34 (20·9)	0·015
Reduced oral intake	4 (2·3)	3 (1·8)	0·750

© 2018 Markar SR, et al. *JAMA Oncology*.

eMethods 9: GC-MS cross platform validation

eTable 10: VOC identification by TD-GC-MS and direct injection SIFT-MS

Compound	TD-GC-MS						SIFT-MS		
	RI	Mass Spectrum (relative intensity %)	SNR	High	Low		CONC	High	Low
Methanol[a]	372	**31(99)**,32(74),29(44)	438	5363	1		450	1170	132
Isoprene [a]	480	**67(99)**,68(72),53(45),39(24)	612	5592	7		256	1856	9
Acrolein [a]	489	**56(99)**,27(95),26(72),55(71)	15	101	nd		88	218	16
Propanal [a]	492	**58(99)**,29(88),28(57)	8	141	1		37	119	10
Acetone [a]	498	44(99),**58(24)**	2675	16515	22		1825	12997	141
Dimethyl Sulphide [a]	502	**62(99)**,47(95),45(40),46(36),61(33),32(22)	22	142	1		570	1584	220
Carbon Disulphide [b]	511	**76(99)**,44(16)	151	447	7		223	641	0
Propanol [a]	576	**31(99)**,29(17),27(16),42(13)	11	99	1		81	182	15
Butanal [a]	596	44(99),43(78),72(73),**41(60)**,27(58),57(25)	4	34	nd		16	74	0
Acetic Acid [a]	675	43(99),45(90),**60(74)**	23	137	4		155	541	3
Butenal [a]	683	41(99),39(89),**70(83)**,69(40)	4	32	1		28	53	4
Butanol [a]	698	**56(99)**,31(98),41(87),43(68)	6	71	1		157	383	28
Pentanal [a]	724	**44(99)**,58(48),29(40),41(30),57(30)	5	30	1		10	52	0
Dimethyl Disulphide [b]	781	**94(99)**,79(57),45(47),61(14)	4	34	1		21	102	0
Propanoic Acid [a]	790	74(99),28(92),45(90),29(83),73(64),**55(26)**	3	16	1		65	214	0
Toluene [a]	799	**91(99)**,92(77),65(12),39(10)	73	291	11		48	100	0
Pentanol [a]	829	42(99),**55(94)**,41(68),70(48),31(36)	1	4	nd		66	143	7
Hexanal [a]	859	44(99),**56(81)**,41(69),57(38),72(16),82(14)	12	42	1		9	25	0
Butyric Acid [a]	900	**60(99)**,73(32),41(16)	4	32	1		47	90	0
m-Xylene [b]	933	**91(99)**,106(52),105(23),77(15)	37	369	6		18	56	0
Heptanal [a]	986	**70(99)**,41(89),44(88),43(83),55(77),96(16)	11	40	2		5	21	0
Pentanoic Acid [a]	1012	**60(99)**,73(35),41(17)	1	4	nd		22	83	0
Octanal [a]	1099	43(99),44(80),41(67),56(65),**84(55)**	20	72	2		5	17	0
Hexanoic Acid [a]	1112	**60(99)**,73(44),41(19)	3	9	1		21	60	0
Phenol [a]	1158	**94(99)**,66(38),65(26),39(24)	54	321	9		8	36	0
Nonanal [a]	1200	**57(99)**,41(89),43(87),56(79),70(42),98(40)	51	206	1		3	23	0
Methyl Phenol [a]	1241	**108(99)**,107(95),79(34),77(32)	4	42	1		20	75	0
Menthol [a]	1260	71(99),81(89),**95(79)**,67(40),55(38),41(35),123(35),138(16)	4	18	nd		4	19	0
Decanal [a]	1291	43(99),57(61),70(47),82(34),**112(22)**	42	211	nd		3	14	0
3- / 4-Ethylphenol [a]	1323	**107(99)**,122(37),77(28),79(15)	4	13	1		12	52	0

Footnote: RI indicates retention index estimated through C4-C20 alkane retention on ZB-624 column; SNR indicates mean of signal-to-noise ratio based on quantification fragment; nd indicates not detected; CONC indicates mean concentration in ppbV; Mass spectrum in bold indicates quantification fragment m/z; [a] indicates GC-MS identification based on matching of NIST library and RI of authentic standard; [b] indicates GC-MS identification based on matching of NIST library only.

© 2018 Markar SR, et al. *JAMA Oncology*.

A previous study [1] depicts that volatile constituents consisting hydrophilic –OH or –COOH functional group tend to adhere to the non-polar column through Van der Waals forces and to each other, resulting lower vapour pressure and undesired chromatograph behaviour such as peak tailing. Non-polar column phase such as ZB-624 column exhibits less suitable for analysis of polar constituents. For a successful GC determination of underivatised free fatty acids, application of polar FFAP column phase would be preferred [2].

Amongst all identified VOC, propanal was distinctly distinguished from the unsaturated acrolein (2-propenal) with resolution over 1.0 on 624 column. In addition, the character ion 58 m/z and 56 m/z for propanal and acrolein respectively is useful for confirmation of their identity. Meanwhile, verification of dimethyl sulphide in GC-MS analysis clarifies the ambiguous SIFT-MS identification of isobaric dimethyl sulphide with ethanethiol as both compounds share similar MS pattern but exhibit different retention properties on this separating column. Nevertheless, neither of ethanethiol was found by GC-MS analysis. Dimethyl sulfide was identified previously as the predominant volatile sulfur compound in breath malodor, which could be a breakdown product of dimethyl sulfoniopropionate. It was also produced by the bacterial metabolism of methanethiol [3]. Apart from 2-ethylphenol, presence of ethylphenol isomer in breath was confined to 3-ethylphenol and 4-ethylphenol GC-MS analysis due to their varying boiling point nature. 3-ethylphenol and 4-ethylphenol were co-eluted in the applied column. Both compounds have been listed in the Human Metabolome Database (http://www.hmdb.ca/) whilst 4-Ethylphenol is a phenolic compound produced in wine and beer by the spoilage yeast *Dekkera bruxellensis* [4] but its presence in breath is yet reported.

© 2018 Markar SR, et al. *JAMA Oncology.*

REFERENCES

1. Poole CF (2012). Packed Columns for Gas-Liquid and Gas-Solid Chromatography in Gas Chromatography, Edited by Poole CF. Elsevier, MA, USA. ISBN 978-0-12-385540-4.

2. Zhao G, Nyman M, Jönsson JA (2006). Rapid determination of short-chain fatty acids in colonic contents and faeces of humans and rats by acidified water extraction and direct-injection gas chromatography, Biomedical Chromatography 20: 674-682.

3. Todd JD, Rogers R, Li YG, Wexler M, Bond PL, Sun L, et al. (2007). Structural and regulatory genes required to make the gas dimethyl sulfide in bacteria. Science 315: 666-669.

4. Schifferdecker AJ, Dashko S, Ishchuk OP and Piškur J (2014). The wine and beer yeast Dekkera bruxellensis. Yeast 31: 323-332.

© 2018 Markar SR, et al. *JAMA Oncology.*

<u>eMethods 10: Comparison of VOCs concentrations between cancer and controls and regression for confounding variables.</u>

eTable 11: Univariate and Multivariate Comparison of concentration of 5 VOCs between cancer and control patients.

VOC	Control group (median (IQR)) (ppbv)	Cancer group (median (IQR)) (ppbv)	P value	Odds ratio (95% confidence interval)	P value
Butyric acid	54·9 (41·5–74·4)	41·6 (29·0–59·4)	0·002	0·98 (0·97 – 0·99)	<0·001
Pentanoic acid	11·1 (8·5–16·4)	9 (5·7–16·4)	0·163	0·95 (0·93 – 0·98)	0·001
Hexanoic acid	9·7 (6·4–12·8)	14·3 (9·8–24·0)	0·024	1·08 (1·04 – 1·11)	<0·001
Butanal	3·5 (1·8–5·8)	3·7 (2·0–7·3)	<0·001	1·07 (1·02 – 1·12)	0·003
Decanal	1·4 (0·6–2·6)	4·2 (2·1–6·7)	<0·001	1·45 (1·28 – 1·63)	<0·001

© 2018 Markar SR, et al. *JAMA Oncology.*

eTable 12: Linear regression analyses, with dependent variable being concentration of each VOC in the model, and independent being the presence of cancer, with regression for confounding demographic variables that differed between groups

VOC	Odds ratio for cancer	95% confidence interval	P value	Other significant variables
Butyric acid	-19·53	-29·80 to -9·27	<0·001	None
Pentanoic acid	1·90	-1·94 to 5·75	0·331	ASA grade, ACE inhibitor
Hexanoic acid	21·46	0·48 to 43·31	0·045	Smoking history
Butanal	3·72	1·28 to 6·16	0·003	Caucasian, Smoking history, ACE inhibitor
Decanal	5·95	3·58 to 8·32	<0·001	None

*Confounding variables included in model; age, sex, Caucasian ethnicity, smoking history, ASA grade, hypertension, liver impairment, statin, beta-blocker and ACE-inhibitor.

eTable 13: Linear regression analyses, with dependent variable being concentration of each VOC in the model, and independent being the presence of cancer, with regression for confounding presenting symptoms that differed between groups

VOC	Odds ratio for cancer	95% confidence interval	P value	Other significant variables
Butyric acid	-10·27	-19·79 to -0·75	0·035	None
Pentanoic acid	2·18	-1·46 to 5·82	0·240	None
Hexanoic acid	23·56	3·10 to 44·01	0·024	None
Butanal	4·02	1·72 to 6·33	0·001	Gastrointestinal bleeding
Decanal	4·56	2·36 to 6·75	<0·001	None

*Confounding presenting symptoms included in model; abdominal pain before eating, abdominal pain after eating, dysphagia, gastrointestinal bleeding and vomiting.

© 2018 Markar SR, et al. *JAMA Oncology.*

eTable 14: Univariate using Mann Whitney-U test of 5 VOCs between oesophageal and gastric cancer patients.

VOC	Oesophageal cancer group (median (IQR)) (ppbv)	Gastric cancer group (median (IQR)) (ppbv)	P value
Butyric acid	41·7 (27·8–58·8)	41·6 (31·0–60·7)	0·585
Pentanoic acid	8·8 (5·3–15·0)	11·7 (6·3–19·1)	0·201
Hexanoic acid	15·6 (10·8–25·4)	12·6 (8·8–22·1)	0·106
Butanal	3·7 (2·3–7·3)	3·5 (2·0–7·4)	0·414
Decanal	4·0 (2·0–6·1)	4·6 (2·2–7·2)	0·344

© 2018 Markar SR, et al. *JAMA Oncology.*

eMethods 11: Diagnostic accuracy of test based upon clinical parameters for NICE guidelines for endoscopy referral.

Methods

Comparison of predicted cancer risk from clinical parameters and actual OGD findings or histology from endoscopic biopsies (reference standard test) was made, and the overall diagnostic accuracy (sensitivity, specificity, and Receiver Operator Characteristic Curve (ROC) analysis) for this clinical parameter test was determined.

NICE symptom included in risk model
Dyspepsia over the age of 55
Dysphagia
Vomiting
Anaemia
Weight loss
Upper abdominal pain
Upper abdominal mass
GI bleeding

© 2018 Markar SR, et al. *JAMA Oncology.*

eFigure 5

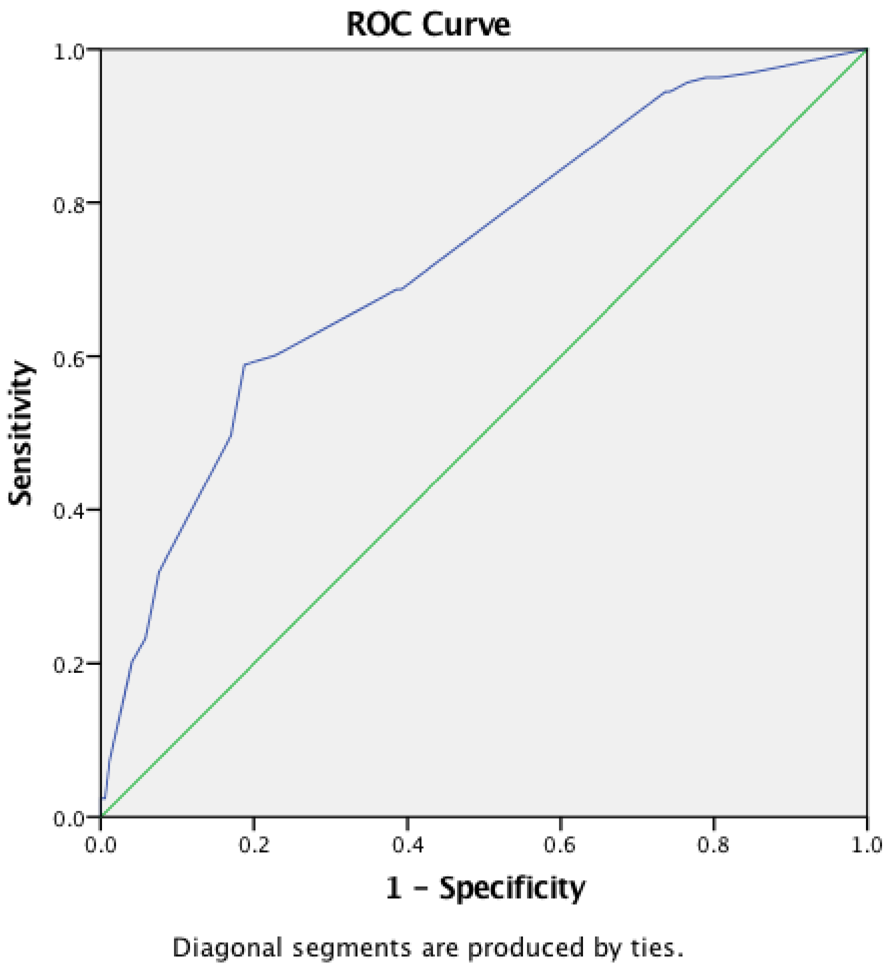

Area Under the Curve

Test Result Variable(s): Predicted probability

Area	Std. Error[a]	Asymptotic Sig.[b]	Asymptotic 95% Confidence Interval	
			Lower Bound	Upper Bound
.729	.027	.000	.675	.782

Sensitivity 59%

Specificity 81%

© 2018 Markar SR, et al. *JAMA Oncology.*

eMethods 12: Decision conferencing – patient pathway

Objective

To solicit the opinion of different stakeholders on the utility of the test and its location in patient pathway.

Methods

Decision conferencing is a series of workshops attended by key players who are facilitated by an impartial specialist in decision theory and group processes, to resolve important issues of concern to the participants. The purposes of decision conferencing are to achieve in the group of key players a shared understanding of the issues, to create a sense of common purpose despite difference of opinion, and to achieve commitment to effective policy and to best practice guidelines. Decision conferencing has been developed over the past 28 years at the London School of Economics by Professor Larry Phillips and his colleagues, and is now used world-wide by hundreds of organisations in all sectors. It is an effective way to tackle difficult problems quickly and thoroughly, and it produces outputs that are readily understood by others because the process of arriving at recommendations is totally transparent.

In applying decision conferencing to this project, we did convene a panel of chairs of multidisciplinary oesophago-gastric teams, gastroenterologists, general practitioners, surgeons and patient members from the oesophageal patient association. The meeting explored the issues, informed participants regarding diagnostic accuracy and cost-effectiveness, and identified factors that affect uncertainty about the location of the test in patient pathway, discussed possible consequences and the key attributes of those consequences, and came to a decision.

© 2018 Markar SR, et al. *JAMA Oncology.*

Results

On 22 January 2015, 18 people (including five patients) gathered at the Royal College of Surgeons to provide guidance for the developers of a breath test for oesophago-gastric cancer that will ensure adequate patient uptake and provide early diagnosis.

Following introductions around the table, data were presented about oesophago-gastric cancer, noting that the incidence of oesophageal adenocarcinoma in the UK is the highest in the world. It was explained that patients in the UK take too long to present to their GP, partly because symptoms of heartburn and indigestion are not widely recognised as potentially indicative of oesophago-gastric cancer. Endoscopy is the gold standard for diagnosis, but it is costly and invasive and experienced as a very unpleasant investigation by many patients.

Furthermore it was explained the status of the breath test approach, with data suggesting that the risk prediction model has a good sensitivity and specificity. We stated that the goals for implementing the breath test are, for patients, earlier diagnosis at an early stage of the disease, better survival, and more patient satisfaction, and, for the NHS, tailored referral and lower cost.

A demonstration of the breath test, using the currently-available technology and software, followed.

Questions were asked about the current status of the breath test, which led to an extended discussion and further questions and answers, as follows:

- Greater general awareness of the disease is needed, and will be stimulated by a programme in the last week of January 2015. If the result is more endoscopies, than that would help to support need for the breath test.

© 2018 Markar SR, et al. *JAMA Oncology.*

- The breath test would be a screening tool providing decision support for the GP, who would take account of other features as well.
- A positive breath test would typically result in the patient being referred for an endoscopy.
- More work is needed on contributing risk factors, such as co-morbidities.
- Pharmacies could alert patients with continuing heartburn. Awareness could be enhanced by pharmacists because they may communicate more amongst themselves than GPs.
- At this stage of development it is not clear if a simple, hand-held device is feasible or desirable.
- Some patients might be too alarmed at a positive finding, but there are soft answers when the breath test is positioned as a screening device, not a programme.
- The group agreed that GP surgeries are the first step in positioning the test. Pharmacies would be second, but only if it would not be necessary (as now) to send off the breath sample to complete the test.

Discussion

The consensus reached through this process of decision conferencing was that at least initially, the breath test for oesophago-gastric cancer would be ideally situated in the primary care setting or general practitioners. The most commonly cited reason for this was that this is the point of primary referral for diagnostic endoscopy, and thus the breath test may be able to triage patients for endoscopy. The benefits identified by the majority of participants would be that the breath test would provide objective criteria that may support decision-making by GPs.

However

© 2018 Markar SR, et al. *JAMA Oncology.*

workshop participants did also suggest that there is a portion of patients who fail to seek medical attention for long-standing symptoms of heartburn, and would typically self-medicate with medications purchased over the counter in a pharmacy. Therefore a secondary position for the breath test may be in the pharmacy in order to reach this population of patients who typically would not seek medical attention.

A further important finding from this workshop was that the majority of participants were unsure as to the optimal patient interface for any breath test in the future. This is clearly needed to plan in the next stage of the breath test development as a robust breath test in clinical practice is unlikely to gain widespread dissemination using breath bags for sample collection. Sensor based technology may allow for the development of disease-specific hand-held devices capable of utilisation in the primary care setting. However participants in the workshop felt that delivery of the results of the test would be critical to reduce the psychological stress upon the patient and therefore a sensor based hand-held device would need to be performed by a trained medical professional capable of delivering the results of the test in a balanced manner. The alternative would be to utilise thermal desorption tubes for breath sample storage, and transport for analysis to a central laboratory [289]. A possible advantage of thermal desorption tubes would be the potential to allow breath profiling of a number of diseases and therefore the test could be more cost-effective and utilised for multiple purposes.

Conclusions

- The ideal position of the breath test in the diagnostic pathway would initially be in the GP surgery and community pharmacy as a secondary location.

- The exact device for breath sampling as a hand-held device to allow for point-of-care testing or thermal desorption tubes to allow for laboratory testing, remains undetermined by the present workshop.

© 2018 Markar SR, et al. *JAMA Oncology.*
